# Risk Factors Analysis of Thoracic Trauma Complicated With Acute Respiratory Distress Syndrome and Observation of Curative Effect of Lung-Protective Ventilation

**DOI:** 10.3389/fsurg.2021.826682

**Published:** 2022-01-24

**Authors:** Xiaoyu Ma, Zefang Dong, Yusuo Wang, Peidong Gu, Jinghua Fang, Shaolin Gao

**Affiliations:** ^1^Department of Thoracic Surgery, The Second Hospital of Hebei Medical University, Shijiazhuang, China; ^2^Ningjin County Maternity and Childcare Hospital of Hebei Province Emergency Surgery, Xingtai, China

**Keywords:** thoracic trauma, acute respiratory distress syndrome, risk factors, lung-protective ventilation, curative effect

## Abstract

**Purpose:**

To explore the risk factors of acute respiratory distress syndrome (ARDS) secondary to thoracic trauma and the therapeutic effect of protective lung ventilation in patients with acute respiratory distress syndrome complicated with thoracic trauma.

**Methods:**

We collected 206 patients with thoracic trauma admitted to our hospital from September 2017 to March 2021, counted the incidence of ARDS and analyzed the risk factors of ARDS. To observe the clinical efficacy of the application of lung-protective ventilation therapy in patients with thoracic trauma combined with ARDS.

**Results:**

Among 206 patients with thoracic trauma, there were 82 cases of combined ARDS, and its incidence was 39.81%. The 82 patients with ARDS were randomly divided into the control group and the observation group with 42 cases each, and different ventilation methods were used for treatment. The results showed that the mechanical ventilation time (MVT) was shorter in the observation group than in the control group, and the incidence of ventilator-associated lung injury (VALI) and case fatality rate (CFR) were lower than those in the control group (*P* < 0.05). Arterial partial pressure of oxygen (Pa0_2_), arterial blood carbon dioxide partial pressure (PaCO_2_), and Oxygenation index (arterial partial pressure of oxygen/Fraction of inspiration O_2_, PaO_2_/FiO_2_) were significantly improved better in both groups after treatment; compared with the control group, patients in the observation group had higher Pa02 levels and lower PaCO_2_ levels at 8 h and 24 h after ventilation (*P* < 0.05). Multivariate analysis revealed that blunt trauma, massive blood transfusion, procalcitonin (PCT) level, tumor necrosis factor-α (TNF-α) level, and acute physiology and chronic health score (APACHE II) were all risk factors for Thoracic trauma with ARDS.

**Conclusion:**

Risk factors for the development of ARDS after thoracic trauma are blunt injuries, massive blood transfusion, high PCT and TNF-α levels, and high APACHE II scores, which can be given active interventions in the early stage of clinical practice to improve patient prognosis. The use of protective lung ventilation therapy can improve the clinical outcome of patients with thoracic trauma combined with ARDS, which is important for improving the ventilation effect and respiratory function of patients.

## Introduction

With the socio-economic development, the incidence of traumatic diseases is on a significant rise and the threat of trauma to human health is becoming more and more prominent. Trauma is now a global health problem and is one of the important causes of death in the young population (1). Thoracic trauma is a common site of involvement when the body is traumatized, and the lungs are the main organ involved in the early onset of severe thoracic trauma patients when they are admitted to the hospital (2). Acute respiratory distress syndrome (ARDS) is an acute diffuse lung injury that occurs within a short period of time and can cause severe hypoxemia leading to hypoxic damage to various organs throughout the body, and is the most common complication in patients with severe trauma (3). The current clinical treatment of severe thoracic trauma combined with acute respiratory distress syndrome mostly uses mechanical ventilation to ensure respiratory support. Therefore, reasonable and effective mechanical ventilation has an important impact on improving the treatment effect and reducing the mortality rate (4). Lung-protective ventilation therapy is a treatment with low tidal ventilation and permissive high carbon dioxide (CO_2_) while meeting the patient's basic oxygenation, which can prevent pulmonary compression injury due to large tidal ventilation (5). Some studies (6, 7) have shown that the application of lun-protective ventilation therapy in the treatment of patients who have thoracic trauma combined with ARDS can achieve better therapeutic results.

Different from ARDS caused by infection, aspiration and other reasons, patients with ARDS secondary to trauma are usually younger, and effective interventions at an early stage of onset can lead to better outcomes and a good patient prognosis (8). Yet the difficulty in salvage lies in the early identification of severe trauma as secondary to ARDS, so screening for predictive risk factors to guide early clinical intervention is necessary (9). In this study, the basic data and pathophysiological characteristics of 206 patients with thoracic trauma treated in our hospital in the past 3 years were collected, and logistic regression models were used to analyze the risk factors for secondary ARDS after thoracic trauma. The clinical effects of different ventilation modalities in patients with ARDS combined with thoracic trauma were analyzed, aiming to provide a reference basis for clinical treatment.

## Information and Methods

### Case Sources and Basic Information

Total of 206 cases of thoracic trauma patients admitted to our hospital from September 2017 to March 2021 were selected as the study subjects. Eighty two patients with severe thoracic trauma combined with ARDS admitted during this period were taken as the main observation objects, and they were divided into 41 cases each in the observation group and the control group by double-blind randomization method, with the control group receiving conventional ventilation treatment and the patients in the observation group receiving lung-protective ventilation protocol. In the control group, 24 males and 17 females were aged from 34 to 60 years, with an average of (47.35 ± 10.37) years. In the observation group, 23 males and 18 females were aged from 35 to 59 years, with an average of (48.29 ± 10.52) years. The differences between the two groups in general data such as gender and age were not statistically significant (*P* > 0.05) and were comparable.

### Diagnostic Criteria

Thoracic trauma (10): ① multiple rib fractures leading to a shackled chest; ② severe pulmonary contusion; ③ moderate or greater haemopneumothorax; ④ tracheobronchial rupture; ⑤ large vessel injury to the heart. The diagnosis can be made on the basis of one of the above.

The diagnosis of ARDS was made according to the Berlin-defined diagnostic criteria for ARDS (11): new onset of dyspnoea or worsening of pre-existing respiratory symptoms within 1 week of a known clinical diagnosis; and an arterial Oxygenation index (arterial partial pressure of oxygen/Fraction of inspiration O_2_, PaO_2_/FiO_2_) <300.

### Inclusion Criteria

 ① All patients met the diagnostic criteria for both thoracic trauma and ARDS and had an injury severity score (ISS) of ≥16, and the diagnosis was made by combining the patient's clinical symptoms, pulmonary infiltrative shadow, and oxygenation index; ② required mechanical ventilation; ③ age ≥ 18 years; ④ the only causative factor was trauma; ⑤ were admitted to the hospital within 24 h of the causative injury and were first seen in our hospital; ⑥ the patient's clinical information was complete and the patient or family had informed the study group and had signed the relevant consent form.

### Exclusion Criteria

 ① patients with a long history of chronic lung disease; ② patients with a history of lung surgery; ③ those with autoimmune diseases; ④ those with infections; ⑤ those with ARDS due to non-traumatic causes or those who have been treated at other hospitals for a longer period of time and then transferred to our hospital; ⑥ those with active acute bleeding conditions.

## Methods

### Treatment Method

First aid and basic treatment of trauma: Upon admission, the patient was thoroughly assessed and the management was tailored to the patient's individual circumstances. For patients with fractures, the ribs were first fixed with chest straps, and then internal fixation was performed after communication with the patient and family and obtaining consent. Patients with hemothorax were treated with thoracal closed drainage. For patients with shock, rehydration and blood transfusion were performed. For patients with inflammatory symptoms, corticosteroids were given to reduce tissue damage and improve microcirculation for 4–7 days and then reduced. Patients were given broad-spectrum antibiotics as necessary to prevent the development of infection. After emergency trauma management, all patients were orotracheally intubated or tracheotomized and connected to a ventilator for assisted breathing, with the control group on conventional ventilation and the observation group on lung protective ventilation.

The ventilator parameters for the conventional ventilation protocol were set as follows: positive end expiratory pressure (PEEP) of 3–10 cmH_2_O, tidal volume (VT) of 10–15 mL/kg, respiratory rate of 15–18 breaths/min, inspiration-exhalation ratio (I:E) = 1:1.5–2.0, frequency was 12–16 breaths/min.

Lung-protective ventilation protocol: low tidal volume ventilation combined with optimal PEEP, and low tidal volume sequential pulmonary resuscitation (RM) when the abdominal pressure (IAP) has basically returned to normal (grade I abdominal hypertension, IAP ≤ 15 mmHg). The parameters were set as PEEP of 5–15 cmH_2_O, I:E = 1:1.0–2.0; VT of 6–8 mL/kg, and frequency of 18–20 breaths/min.

### Observed Indicators

The treatment status such as mechanical ventilation time (MVT), ventilator-associated lung injury (VALI) incidence and case fatality rate (CFR) were collected from both groups. The blood gas analysis indexes such as PaO_2_, arterial blood carbon dioxide partial pressure (PaCO_2_) and PaO_2_/FiO_2_ were observed and recorded at different times before and after treatment.

The gender, age, body mass index (BMI), causes of trauma, nature of injury, massive blood transfusion, acute physiology and chronic health score (APACHE II), ISS Score were collected from 206 patients with thoracic trauma. In addition, the following index were used: underlying disease, history of long-term smoking, history of alcohol abuse, serum C-reactive protein (CRP), procalcitonin (PCT), interleukin-6 (IL-6) and tumor necrosis factor-alpha (TNF-α) levels within 1 week of admission. We compared the differences in the above indicators between patients who had a combined ARDS and those who did not have a combined ARDS in thoracic trauma, and used logistic regression to analyze the risk factors for combined ARDS in patients with thoracic trauma.

### Statistical Methods

SPSS 22.0 software was used for data processing, and the measurement data were expressed as mean ± standard deviation (Mean ± SD), the *t*-test was used for two-by-two comparisons. The count data were expressed as (*n*, %) and the chi-square (χ^2^) test was used. Logistic regression model was used for multi-factor analysis of thoracic trauma combined with ARDS. *P* < 0.05 was considered as a statistically significant difference.

## Results

### Univariate Analysis of Thoracic Trauma in Combination With ARDS

Eventually, statistics revealed that 82 of 206 patients with thoracic trauma had combined ARDS, with an incidence of 39.81%; 124 patients without combined ARDS (60.29%). The basic data of patients with thoracic trauma in different conditions were analyzed, and the results showed that there were differences between patients with combined ARDS and those without combined ARDS in long-term smoking, nature of injury, massive blood transfusion, APACHE II score, ISS score, CRP, PCT, IL-6, TNF-α, etc. (*P* < 0.05). There were no differences in gender, age, BMI, causes of trauma, alcoholism and underlying diseases were not different (*P* > 0.05) (Table 1). This suggests that long-term smoking, blunt injuries, massive blood transfusion, high APACHE II, ISS score, high CRP, PCT, IL-6, and TNF-α levels may be associated in patients with thoracic trauma secondary to ARDS.

**Table 1:** Univariate analysis of thoracic trauma combined with ARDS.

**Influencing factors**		**Merged ARDS (*n =* 82)**	**Unconsolidated ARDS (*n =* 124)**	***t* value or *χ^2^* value**	***P-*value**
Gender (*n*, %)	Male	47 (57.32)	69 (55.65)	0.056	0.813
	Female	35 (42.68)	55 (44.35)		
Age (years, Mean ± SD)		47.93 ± 10.45	45.69 ± 8.76	1.588	0.114
BMI (kg/m^2^, Mean ± SD^)^		25.26 ± 4.26	25.19 ± 4.03	0.119	0.905
Causes of trauma (*n*, %)	Traffic Accidents	31 (37.80)	43 (34.68)	1.026	0.906
	Sharp objects	15 (18.29)	26 (20.97)		
	Falling	19 (23.17)	25 (20.16)		
	Crushing	12 (14.63)	19 (15.32)		
	Explosion	5 (6.10)	11 (8.87)		
Alcoholism (*n*, %)	Yes	20 (24.39)	38 (30.65)	0.955	0.329
	No	62 (75.61)	86 (69.35)		
Long-term smoking (n,%)	Yes	40 (48.78)	42 (33.87)	4.579	0.032
	No	42 (51.22)	82 (66.13)		
Underlying disease (*n*, %)	Diabetes	22 (26.83)	35 (28.23)	0.048	0.826
	High blood pressure	17 (20.73)	28 (22.58)	0.099	0.753
	Coronary heart disease	11 (13.41)	25 (20.16)	1.556	0.212
Nature of injury (*n*, %)	Blunt injury	57 (69.51)	44 (35.48)	22.870	<0.001
	Penetrating injury	25 (30.49)	80 (64.52)		
Massive blood transfusion (*n*, %)	Yes	37 (45.12)	32 (25.81)	8.267	0.004
	No	45 (54.88)	92 (74.19)		
APACHE II score (points, Mean ± SD)		22.26 ± 3.02	20.13 ± 2.41	5.607	<0.001
ISS score (points, Mean ± SD)		21.74 ± 3.25	20.86 ± 2.98	2.001	0.047
CRP (mg/L, Mean ± SD)		153.32 ± 31.25	138.59 ± 22.28	3.948	<0.001
PCT (×10^9^/L, Mean ± SD)		28.47 ± 5.96	16.16 ± 2.04	13.572	<0.001
IL-6 (ng/L, Mean ± SD)		36.59 ± 11.04	27.52 ± 8.38	6.690	<0.001
TNF-α (ng/L, Mean ± SD)		46.74 ± 12.25	36.19 ± 11.77	6.196	<0.001

### Multi-Factor Analysis of Thoracic Trauma Combined With ARDS

Whether thoracic trauma patients had combined ARDS was used as the dependent variable (yes = 1, no = 0), and the relevant factors with statistically significant differences in the univariate analysis were used as independent variables for multifactor logistic regression analysis (Multi-factor assignments were shown in Table 2), which showed that blunt injury, massive blood transfusion, APACHE II score, PCT level, and TNF-α level were all independent risk factors affecting thoracic trauma patients with independent risk factors for secondary ARDS in patients with thoracic trauma (*P* < 0.05, Table 3).

**Table 2:** Assignment for multivariate analysis of factors.

**Factors**	**Assignment**
Blunt injury	Yes = 0, No = 1
Massive blood transfusion	Yes = 0, No = 1
APACHE II score	>25 = 0, 15–25 = 1, <15 = 2
PCT	>30 = 0, 15–30 = 1, <15 = 2
TNF-α	>60 = 0, 40–60 = 1, 20~40 = 2, <20 = 3

**Table 3:** Multifactorial analysis of ARDS in combination with thoracic trauma.

**Influencing factors**	***B***	***SE***	***Waldχ^2^***	**OR value**	**95%CI**	***P*-value**
Blunt injury	0.937	0.322	6.012	2.552	1.358–4.798	0.013
Massive blood transfusion	1.426	0.922	9.343	4.162	1.631–10.622	0.003
APACHE II score	0.712	0.250	11.324	2.038	1.249–3.327	0.001
PCT	0.878	0.305	6.352	2.406	1.323–4.375	0.017
TNF-α	1.254	0.530	5.235	3.504	1.240–9.903	0.031

*Note: B is the regression coefficient, SE is the standard error, Wald is the test statistic, OR is the dominance ratio, and 95% CI is the 95% confidence interval*.

### Comparison of Clinical Efficacy

**Figure 1:**
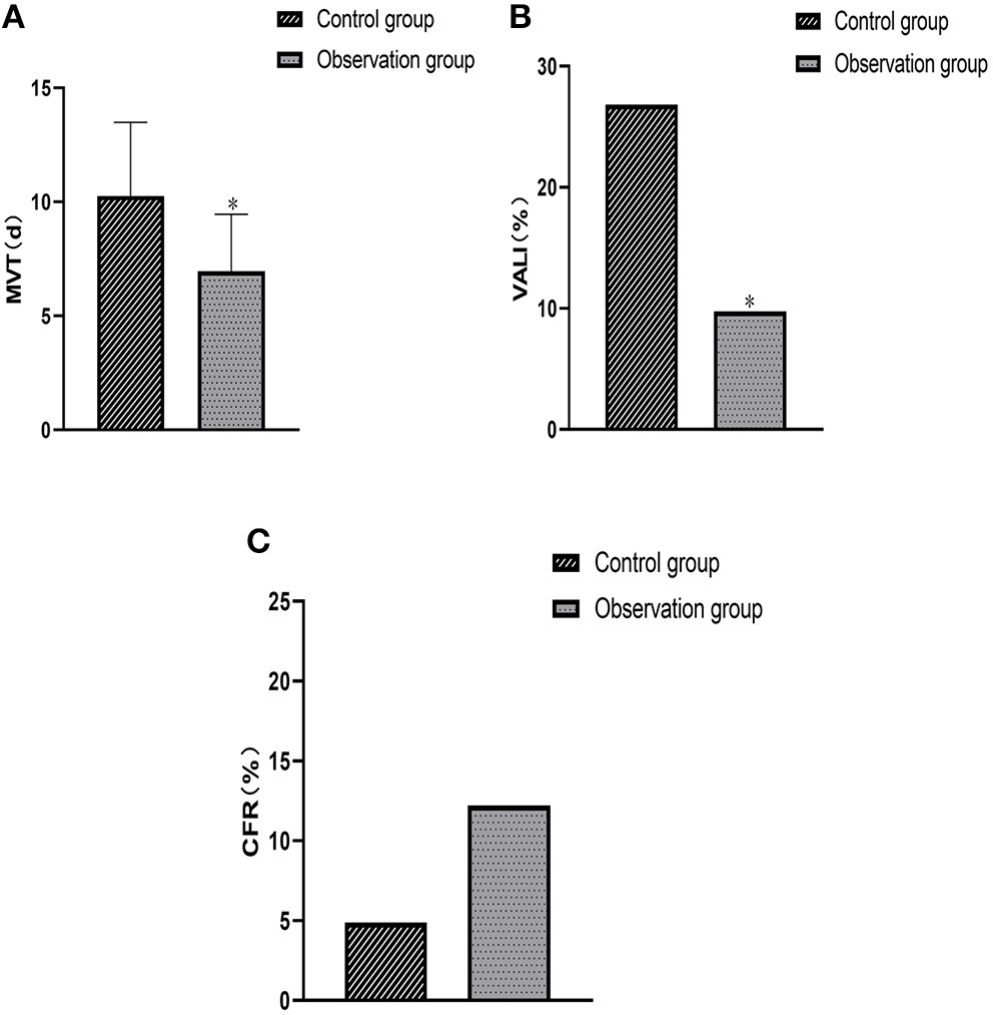
Comparison of clinical efficacy between the two groups. **(A)** Comparison of average MVT. **(B)** Comparison of the occurrence of VALI. **(C)** Comparison of CFR. Compared with the control group, ^*^P < 0.05.

After different ventilation treatments in the two groups, the time to MVT, incidence of VALI and CFR were (10.26 ± 3.23) days, 26.83% (11/41) and 4.88% (2/41) in the control group, respectively; and (6.95 ± 2.51) days, 9.76% (4/41) and 12.20% (5/41) in the observation group, respectively. After analysis, the results showed that the MVT time in the observation group was shorter than that in the control group, and the incidence of VALI was lower than that in the control group (*P* < 0.05); the CFR in the observation group was slightly lower than that in the control group, although the difference between the two groups was not statistically significant (*P* > 0.05) (Figure 1).

### Comparison of Blood Gas Analysis and Oxygenation Index Between the Two Groups Before and After Treatment

**Figure 2:**
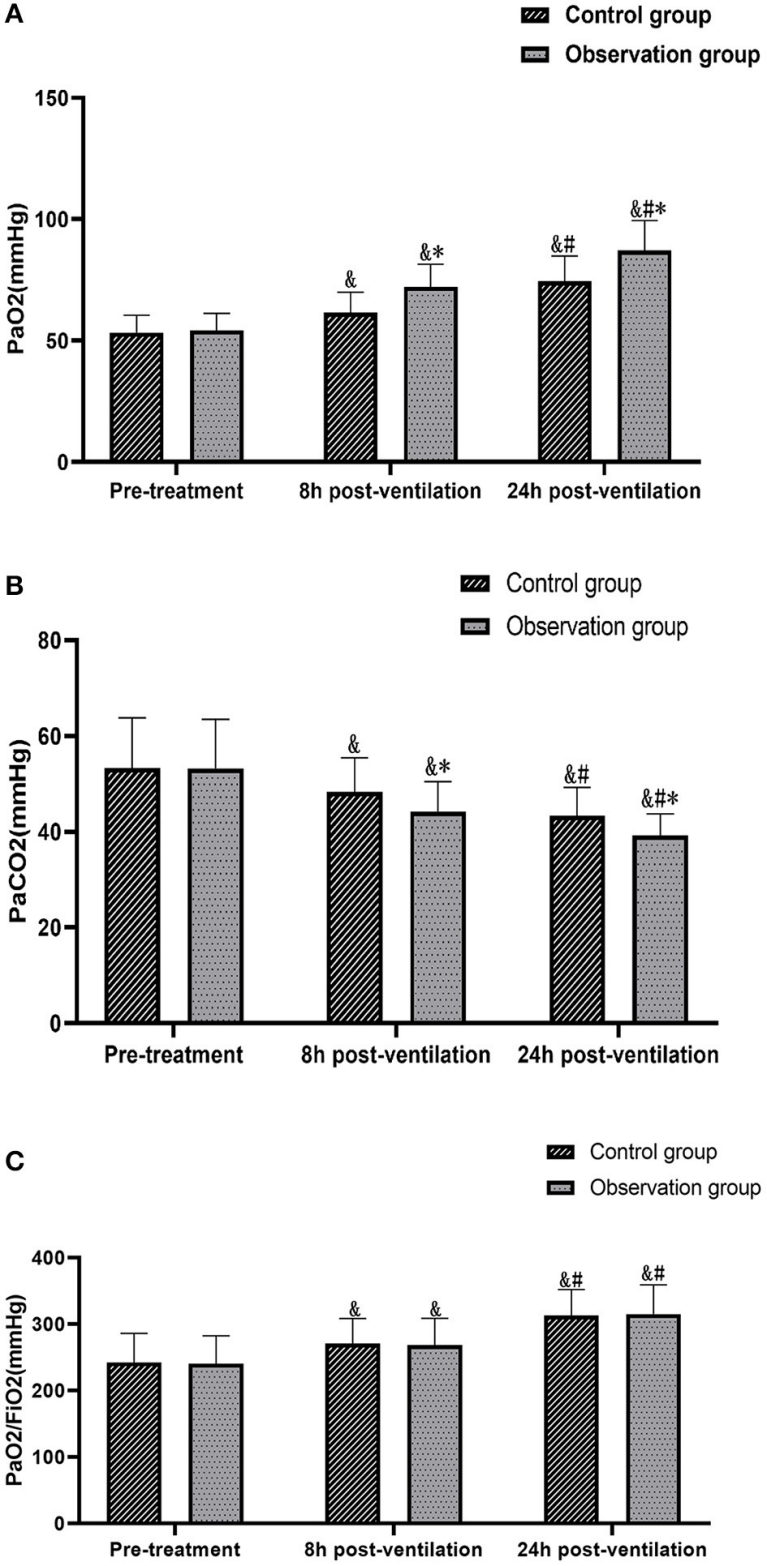
Comparison of the changes in blood gas analysis values and oxygenation index between the two groups at different times. **(A)** Comparison of PaO_2_. **(B)** Comparison of PaCO_2_. **(C)** Comparison of PaCO_2_/FiO_2_. Compared with the same group pre-treatment, ^&^*P* < 0.05. Compared with the same group 8 h post-ventilation, ^#^*P* < 0.05. Comparison with control group over the same period, ^*^P <0.05.

Comparison of the blood gas analysis and oxygenation index data collected from the two groups before treatment and at different times after ventilation showed no statistically significant differences in PaO_2_, PaCO_2_ and PaO_2_/FiO_2_ between the two groups before treatment (*P* > 0.05). PaO_2_, PaCO_2_ and PaO_2_/FiO_2_ at 8 h and 24 h after ventilation were higher than those before treatment in both groups, and the levels of PaO_2_ and PaCO_2_ detected at the same time points after treatment were significantly higher in the observation group than in the control group (*P* < 0.05); the difference in PaO_2_/FiO_2_ between the two groups at different time points after ventilation was not statistically significant (*P* > 0.05) (Figure 2).

## Discussion

Severe trauma is always a major problem in clinical treatment, among which the chest is a common site of trauma. Severe trauma to the patient's chest from multiple causes can often cause direct damage to the lungs, and if the trauma does not receive timely and effective intervention, the patient's condition can further develop into acute hypoxic respiratory failure, shock, and other symptoms, eventually leading to the development of ARDS (12, 13). However, the early recognition rate of severe thoracic trauma combined with ARDS is currently not high in clinical practice, so there is an urgent need to screen for independent risk factors for thoracic trauma combined with ARDS to guide early recognition and effective interventions (14, 15).

The results of this study showed that blunt injuries, massive blood transfusion, and APACHE II score were all independent risk factors for combined ARDS in patients with thoracic trauma and had some predictive value for ARDS secondary to thoracic trauma. Blunt injuries represent a significant proportion of traumatic injuries, and some studies (16) have pointed out their relationship with the development of ARDS. When a blunt blow occurs in the chest, the strong external force can cause rib fractures that can damage the integrity of the thorax or even produce a flail chest, and is often accompanied by hemothorax and pneumothorax at an early stage, leading to respiratory and circulatory dysfunction and then develop progressive dyspnea and refractory hypoxemia (17, 18). The infusion of a large number of blood products can induce inflammatory cells to activate in the lungs and secrete more inflammatory mediators, leading to capillary leakage and lung injury which in turn leads to ARDS (19). The APACHE II scoring system is used to evaluate the patient's condition based on the patient's vital signs and biochemical indicators, and an excessively high APACHE II score within 24 h of admission usually indicates that the patient's respiratory function is already significantly impaired (20). Moreover, the damage of trauma is mostly progressive, especially in patients with thoracic trauma there are mostly pulmonary contusions, which are accompanied by a gradual increase in exudation and a further decrease in pulmonary ventilation aggravating hypoxemia, leading to the rapid development of ARDS (21). The results also showed that inflammatory cells such as PCT and TNF-α were also independent risk factors for ARDS secondary to thoracic trauma. To analyze the reasons for this, vascular endothelial damage after severe thoracic trauma can cause a large release of thrombomodulin (TM) and participate in regulating the coagulation process of the body, leading to microthrombus formation in the lung, microcirculatory blockage and structural damage, inducing pulmonary capillary spasm and increased permeability, activating inflammatory cells, and thus inducing the development of ARDS (22).

The results of this study showed that lung-protective ventilation therapy was more advantageous in improving oxygenation indices and protecting lung function in patients suffering from thoracic trauma combined with ARDS, and was able to reduce the duration of mechanical ventilation and the incidence of VALI in patients with a very low morbidity and mortality rate. At the present stage, ventilator-assisted ventilation is often used to treat patients with Thoracic trauma combined with ARDS on the basis of symptomatic treatment, which can precisely control oxygen therapy to avoid ventilator fatigue and obtain satisfactory treatment results (23). For the traditional conventional ventilation in clinical practice, patients are subjected to high airway pressure due to the large ventilation volume, which predisposes them to lung air pressure injury and thus has a serious impact on their prognosis. Lung-protective ventilation treatment can be used clinically according to the patient's specific situation, and this ventilation method with a lower tidal volume of 6–8 ml/kg and optimal PEEP can avoid overinflation and expansion of lung tissue, shorten expiratory time, ensure adequate ventilation, and help reduce the incidence of VALI (24).

Inconclusion, blunt injury, massive blood transfusion, APACHE II score, PCT, and TNF-α level are all risk factors for the occurrence of Thoracic trauma combined with ARDS, and early identification of these high-risk factors to guide clinical adoption of anticipatory treatment measures is expected to reduce the incidence of ARDS combined with Thoracic trauma. Lung-protective ventilation therapy is important to improve the ventilation effect of patients, reduce the occurrence of VALI and improve respiratory function.

## Data Availability Statement

The original contributions presented in the study are included in the article/supplementary material, further inquiries can be directed to the corresponding author/s.

## Ethics Statement

The studies involving human participants were reviewed and approved by the Ethics Committee of The Second Hospital of Hebei Medical University. Written informed consent to participate in this study was provided by the participants' legal guardian/next of kin.

## Author Contributions

XM, ZD, YW, PG, and JF: collected the clinical datas, analyzed, and counted the datas. XM: wrote the manuscript. SG: modified the language and he is the Corresponding author. All authors contributed to the article and approved the submitted version.

## Conflict of Interest

The authors declare that the research was conducted in the absence of any commercial or financial relationships that could be construed as a potential conflict of interest.

## Publisher's Note

All claims expressed in this article are solely those of the authors and do not necessarily represent those of their affiliated organizations, or those of the publisher, the editors and the reviewers. Any product that may be evaluated in this article, or claim that may be made by its manufacturer, is not guaranteed or endorsed by the publisher.

## References

[1] Li L Liang J Fu H. An update on the association between traumatic brain injury and Alzheimer's disease: focus on tau pathology and synaptic dysfunction. Neurosci Biobehav Rev. (2021) 120:372–86. 10.1016/j.neubiorev.2020.10.020 33171143 PMC9725170

[2] Dogrul BN Kiliccalan I Asci ES Peker SC. Blunt trauma related chest wall and pulmonary injuries: an overview. Chin J Traumatol. (2020) 23:125–38. 10.1016/j.cjtee.2020.04.003 32417043 PMC7296362

[3] Thompson BT Chambers RC Liu KD. Acute Respiratory Distress Syndrome. N Engl J Med. (2017) 377:562–72. 10.1056/NEJMra1608077 28792873

[4] Bertoni M Spadaro S Goligher EC. Monitoring patient respiratory effort during mechanical ventilation: lung and diaphragm-protective ventilation. Crit Care. (2020) 24:106. 10.1186/s13054-020-2777-y 32204729 PMC7092676

[5] Abrams D Schmidt M Pham T Beitler JR Fan E Goligher EC. Mechanical ventilation for acute respiratory distress syndrome during extracorporeal life support. Res Pract Am J Respir Crit Care Med. (2020) 201:514–25. 10.1164/rccm.201907-1283CI 31726013

[6] Guérin C Papazian L Reignier J Ayzac L Loundou A Forel JM. investigators of the Acurasys and Proseva trials. Effect of driving pressure on mortality in ARDS patients during lung protective mechanical ventilation in two randomized controlled trials. Crit Care. (2016) 20:384. 10.1186/s13054-016-1556-2 27894328 PMC5126997

[7] O'Gara B Talmor D. Perioperative lung protective ventilation. BMJ. (2018) 362:k3030. 10.1136/bmj.k3030 30201797 PMC6889848

[8] Jabaudon M Blondonnet R Constantin JM ARDS. in patients with chest trauma: better safe than sorry. Anaesth Crit Care Pain Med. (2019) 38:221–2. 10.1016/j.accpm.2019.04.006 31076142

[9] de Roulet A Burke RV Lim J Papillon S Bliss DW Ford HR. Pediatric trauma-associated acute respiratory distress syndrome: Incidence, risk factors, and outcomes. J Pediatr Surg. (2019) 54:1405–10. 10.1016/j.jpedsurg.2018.07.005 30041860

[10] Baker SP O'Neill B Haddon W Long WB. The injury severity score: a method for describing patients with multiple injuries and evaluating emergency care. J Trauma. (1974) 14:187–96. 10.1097/00005373-197403000-00001 4814394

[11] ARDS Definition Task Force Ranieri VM Rubenfeld GD Thompson BT Ferguson ND Caldwell E. Acute respiratory distress syndrome: the Berlin Definition. JAMA. (2012) 307:2526–33. 10.1001/jama.2012.5669 22797452

[12] Fu PK Wu CL Tsai TH Hsieh CL. Anti-inflammatory and anticoagulative effects of paeonol on LPS-induced acute lung injury in rats. Evid Based Complement Alternat Med. (2012) 2012:837513. 10.1155/2012/837513 22454687 PMC3291481

[13] Ramin S Charbit J Jaber S Capdevila X. Acute respiratory distress syndrome after chest trauma: Epidemiology, specific physiopathology and ventilation strategies. Anaesth Crit Care Pain Med. (2019) 38:265–76. 10.1016/j.accpm.2018.09.009 30342102

[14] Winkelmann M Clausen JD Graeff P Schröter C Zeckey C Weber-Spickschen S. Impact of accidental hypothermia on pulmonary complications in multiply injured patients with blunt chest trauma—a matched-pair analysis. In Vivo. (2019) 33:1539–45. 10.21873/invivo.11634 31471402 PMC6755018

[15] Daurat A Millet I Roustan JP Maury C Taourel P Jaber S. Thoracic Trauma Severity score on admission allows to determine the risk of delayed ARDS in trauma patients with pulmonary contusion. Injury. (2016) 47:147–53. 10.1016/j.injury.2015.08.031 26358517

[16] Mommsen P Zeckey C Andruszkow H Weidemann J Frömke C Puljic P. Comparison of different thoracic trauma scoring systems in regards to prediction of post-traumatic complications and outcome in blunt chest trauma. J Surg Res. (2012) 176:239–47. 10.1016/j.jss.2011.09.018 22099585

[17] Moazed F Hendrickson C Conroy A Kornblith LZ Benowitz NL Delucchi K. Cigarette smoking and ARDS after blunt trauma: the influence of changing smoking patterns and resuscitation practices. Chest. (2020) 158:1490–8. 10.1016/j.chest.2020.05.603 32574574 PMC7545485

[18] Kalbitz M Karbach M Braumueller S Kellermann P Gebhard F Huber-Lang M. Role of complement C5 in experimental blunt chest trauma-induced septic acute lung injury (ALI). PLoS ONE. (2016) 11:e0159417. 10.1371/journal.pone.0159417 27437704 PMC4954719

[19] Robinson BRH Cohen MJ Holcomb JB Pritts TA Gomaa D Fox EE. PROPPR Study Group. Risk factors for the development of acute respiratory distress syndrome following hemorrhage. Shock. (2018) 50:258–64. 10.1097/SHK.0000000000001073 29194339 PMC5976504

[20] Navarrete-Navarro P Rivera-Fernández R Rincón-Ferrari MD García-Delgado M Muñoz A Jiménez JM. GITAN multicenter project. Early markers of acute respiratory distress syndrome development in severe trauma patients. J Crit Care. (2006) 21:253–8. 10.1016/j.jcrc.2005.12.012 16990093

[21] Watkins TR Nathens AB Cooke CR Psaty BM Maier RV Cuschieri J. Acute respiratory distress syndrome after trauma: development and validation of a predictive model. Crit Care Med. (2012) 40:2295–303. 10.1097/CCM.0b013e3182544f6a 22809905 PMC3400931

[22] Ehrnthaller C Flierl M Perl M Denk S Unnewehr H Ward PA. The molecular fingerprint of lung inflammation after blunt chest trauma. Eur J Med Res. (2015) 20:70. 10.1186/s40001-015-0164-y 26303896 PMC4548898

[23] Qi Y. Clinical study on VATS combined mechanical ventilation treatment of ARDS secondary to severe chest trauma. Exp Ther Med. (2016) 12:1034–8. 10.3892/etm.2016.3355 27446317 PMC4950469

[24] Dianti J Matelski J Tisminetzky M Walkey AJ Munshi L Del Sorbo L. Comparing the effects of tidal volume, driving pressure, and mechanical power on mortality in trials of lung-protective mechanical ventilation. Respir Care. (2021) 66:221–7. 10.4187/respcare.07876 32843513

